# Sorption–Desorption Behavior of Doxycycline in Soil–Manure Systems Amended with Mesquite Wood Waste Biochar

**DOI:** 10.3390/plants10122566

**Published:** 2021-11-24

**Authors:** Mohammad I. Al-Wabel, Munir Ahmad, Hamed A. Al-Swadi, Jahangir Ahmad, Yassir Abdin, Adel R. A. Usman, Abdullah S. F. Al-Farraj

**Affiliations:** 1 Soil Sciences Department, College of Food & Agricultural Sciences, King Saud University, P.O. Box 2460, Riyadh 11451, Saudi Arabia; amunir@ksu.edu.sa (M.A.); h.alswadi@hotmail.com (H.A.A.-S.); jahangirsoomra@gmail.com (J.A.); yassirabdin@gmail.com (Y.A.); adosman@ksu.edu.sa (A.R.A.U.); sfarraj@ksu.edu.sa (A.S.F.A.-F.); 2 Department of Soil, Water and Environment, Faculty of Agriculture, Sana’a University, Sana’a 31220, Yemen; 3 Department of Soils and Water, Faculty of Agriculture, Assiut University, Assiut 71526, Egypt

**Keywords:** desorption, ecosystem health, soil contamination, transport, veterinary antibiotics

## Abstract

Elevated levels of doxycycline (DC) have been detected in the environment due to its extensive utilization as a veterinary antibiotic. Sorption–desorption behavior of DC in soil affects its transport, transformation, and availability in the environment. Thus, sorption–desorption behavior of DC was explored in three soils (S1, S2, and S3) after manure application with and without mesquite wood-waste-derived biochar (BC) pyrolyzed at 600 °C. Sorption batch trials demonstrated the highest DC sorption in soil S1 as compared to S2 and S3, either alone or in combination with manure or manure + BC. Chemical sorption and pore diffusion were involved in DC sorption, as indicated by the kinetic models. Soil S1 with manure + BC exhibited the highest Langmuir model predicted sorption capacity (18.930 mg g^−1^) compared with the other two soils. DC sorption capacity of soils was increased by 5.0–6.5-fold with the addition of manure, and 10–13-fold with BC application in a soil–manure system. In desorption trials, manure application resulted in 67%, 40%, and 41% increment in DC desorption in soil S1, S2, and S3, respectively, compared to the respective soils without manure application. In contrast, BC application reduced DC desorption by 73%, 66%, and 65%, in S1, S2, and S3, respectively, compared to the soils without any amendment. The highest DC sorption after BC application could be due to H bonding, π–π EDA interactions, and diffusion into the pores of BC. Hence, mesquite wood-waste-derived BC can effectively be used to enhance DC retention in contaminated soil to ensure a sustainable ecosystem.

## 1. Introduction

Antibiotics have been utilized for decades in large quantities in livestock farming; however, their fate and consequences are still not fully explored [1,2]. Despite the ban on the use of antibiotics as growth-promoting agents in livestock farming in the European Union (Regulation 1831/2003) and the USA (Guidance 213, Veterinary Feed Directive (VFD, Federal Register 2015)), large amounts of these substances are still being prescribed in order to cure bacterial diseases in animal husbandry [3]. Extensive use of antibiotics is posing a serious threat to environmental health, as a significant amount of antibiotic residues is being introduced to the aquatic system through animal feces disposal, dumping of medical waste, and through feeding jettison [1,4]. The use of antibiotics in the livestock industry increased to 63,151 tons in the year 2010, which is expected to be raised by 67% in the year 2030 [5]. The total annual consumption of antibiotics in the agriculture sector is 63,000 to 240,000 tons worldwide [6]. Generally, antibiotics are water soluble and, therefore, approximately 90% of the applied antibiotics are excreted in urine and more than 75% via feces [7]. As a result, the consumed antibiotics cannot be fully absorbed in the animal’s body and about 30–90% of the original compound or its bioactive species are excreted out [4]. Moreover, antibiotic metabolites can transfer to the parent compound, as they are bioactive after excretion. As a consequence, the dosed antibiotics may be excreted to the environment in active form.

Spreading animal manure and slurry as fertilizers is the major cause of soil contamination with veterinary antibiotics. Generally, manure is used in arable soil, where these veterinary antibiotics can leach from surfaces to groundwater [1]. Moreover, antibiotic residues in the soil exert pressure on the native bacterial community and favor the development of antibiotic-resistant bacteria and antibiotic-resistant genes [8,9]. Irrigating the field with antibiotic-laden water, as well as aerial transportation, may also result in soil contamination [10]. Chemical, physical, and biological degradation and adsorption–desorption processes determine the fate of antibiotics in the soil, as well as their transport and retention behavior. However, adsorption and desorption processes are dependent on soil properties and antibiotic characteristics. 

Doxycycline (DC) belongs to the tetracycline (TC) family and it is used against Gram-negative and Gram-positive bacteria. According to the U.S. Food and Drug Administration, the consumption of TC is much higher (approximately 5,866,588 kg) as compared to the other commonly consumed antibiotics [11,12]. Consequently, large quantities of TC have been detected in various environmental compartments, such as soil (86–199 ug kg^−1^), surface water (5.7–88.7 ng L^−1^), drinking water (87–97 ng L^−1^), and ground water (4.4–9.3 ng L^−1^) [12,13,14,15]. A considerable concentration of the DC residues was detected in pig manure and digestates of biogas plants as 381 and 10.5 mg kg^−1^ dry weight, respectively [16]. Likewise, a higher concentration (22.76 mg kg^−1^) of DC was also detected in the swine manure sampled from farms and manure pits in West Flanders, Belgium [17]. The presence of such higher concentrations of DC in the environment is posing serious threats to the environmental sustainability. Thus, finding an economical, efficient, and ecofriendly technique is the main point of concern to environmentalists worldwide. Therefore, investigating the sorption–desorption behavior of DC in the manure-amended soil is very important to study its immobilization, transportation, ecotoxicology, degradation, and bioaccumulation behavior. 

Various technologies, including photodegradation, membrane separation, electrochemistry, Fenton-like, and adsorption, have been employed for the removal of DC from various environmental matrices [18]. However, the majority of these technologies are either expensive or inefficient [19]. Adsorption is one of the well-known techniques for removing organic and inorganic contaminants from the environment, as it is a cost-effective, environmentally friendly, easy, and efficient method. It is considered as one of the significant processes through which DC can be removed from the environment [20]. Previously, various adsorbents, including biochar, activated carbon, chitosan, and nano-metal oxides, have been used as adsorptive materials [21,22]. The DC molecule contains multiple functional groups, including phenol, alcohol, and amino, and can exist as a cation, anion, and zwitterion depending on the pH [23]. Therefore, the use of carbon-based materials as adsorptive agents to remove DC from the environment has attracted significant attention of researchers nowadays [24,25]. These materials contain high adsorption and removal capacities for targeted pollutants. For instance, biochar, which is a porous material derived from waste, has been proved as an efficient and green method for removing a range of organic and inorganic pollutants [26]. Biochar contains a higher surface area, a range of functional groups, and higher sorption affinity for a range of environmental contaminants, and thus has been reported as a low-cost adsorbent for the removal of different types of pollutants, including dyes, heavy metals, pesticides, and antibiotics, from the soil [27,28,29]. However, the characteristics and performance of biochar mainly depend on pyrolytic conditions and feedstock type. For instance, a higher pH of biochar may enhance the metal-binding capacity of biochar [30], while it reduces the affinity to bind DC due to the anionic nature of DC molecule at higher pH levels. Thus, the sorption–desorption behavior of biochar is largely dependent on its characteristics, which are impacted by the nature of feedstock, pyrolysis temperature, and resident time [31]. 

Mesquite (*Prosopis juliflora*), which is an invasive tree, is responsible for generating large volumes of biomass wastes in arid and semi-arid regions of the world [32]. Therefore, recycling this biomass to produce biochar would not only reduce surface pollution, but could help in remediating the contaminated soils [31]. Hence, it was hypothesized that mesquite wood-waste-derived biochar application in soil may enhance the retention of DC through sorption onto its matrix. Hence, the current study was conducted to investigate sorption–desorption behavior of DC in three different soil types amended with manure and manure + mesquite wood-waste-derived biochar via kinetics and equilibrium sorption batch trials. Various kinetics and isotherm models were employed to the sorption data to understand the sorption process. Moreover, the effects of soil types on DC retention and desorption were studied in soil with and without the addition of manure and biochar. 

## 2. Results and Discussion 

### 2.1. Physiochemical Characteristics of Soil and Manure

Collected soil and manure samples were analyzed for their physiochemical characteristics (Table 1). Based on characteristics, soils were named as S1 (pH 7.41), S2 (pH 8.32), and S3 (pH 8.48). Soil S1 was pronounced with sandy clay loam, while S2 and S3 were sandy loam in texture. Soil S1 was slightly alkaline (pH 7.41), whereas S2 and S3 were moderately alkaline with pH of 8.32 and 8.48, respectively. Soil S1 showed a relatively higher electrical conductivity (EC) of 4.82 dS m^−1^ as compared to S2 and S3. Owing to the higher percentage of sand in soil samples, a very low percentage of organic matter was anticipated (0.67–0.87%); however, a better range of cation exchange capacity (CEC) of soil samples, especially S1 (26.39 cmol kg^−1^), suggested suitability of the soils for agricultural activities. Soil S1 contained 8.55% of CaCO_3_, while S2 and S3 showed 9.86% and 9.33% of CaCO_3_, respectively. Lower available phosphorus (P) contents in S2 (8.68 mg kg^−1^) and S3 (7.81 mg kg^−1^) could be due to precipitation and fixation processes owing to higher pH and CaCO_3_ contents as compared to S1 [33]. As anticipated, manure samples exhibited higher organic matter contents (10.34%) and EC (6.61 dS m^−1^), while the pH was slightly alkaline (7.72). The high CEC value of manure could be due to a great number of oxygen-containing functional groups in its composition. On the other hand, a potentially higher EC value showed the presence of supplementary soluble salts in manure samples. Manure was found enriched in available macronutrients, such as P, potassium (K), and sodium (Na), which might be due to added supplements in the feed consumed by the animals. The higher moisture contents (13.03%) were found in manure, while the volatiles and fixed carbon contents in manure were 38.72% and 19.29%, respectively. The presence of higher ash contents (28.96%) in the manure samples suggested the occurrence of mineral compounds [34].

### 2.2. Biochar Characterization 

The proximate characteristics and elemental composition of the produced biochar (BC) are shown in Table 2. The yield of BC produced at 600 °C pyrolysis temperature was recorded as 30.2%, which indicated dehydration and degradation of organic compounds (cellulose, hemicellulose, and lignin) and surface functional groups in biomass (BM). As described in Table 2, a sharp drop in moisture and volatiles was noted in BC due to thermal degradation and decomposition of BM. In contrast, the mineral components of BM were concentrated and condensed with thermal treatment, which resulted in higher ash contents of BC [26]. Additionally, the higher contents of fixed carbon (75.8%) in BC against raw BM (22.6%) showed a great carbon stability, higher aromaticity, and enhanced recalcitrant potential of BC over BM. Elemental composition of BC showed a significant decrease in O and H contents, whereas C contents were further concentrated, which was also supported by proximate analysis. Reduction in O and H contents was mainly due to the enhancement in the loss of volatile materials, dehydration of O–H groups, and further degradation of other surface functional groups, along with replacement by aromatic compounds [35]. Furthermore, the effects of pyrolysis temperature on molar elemental ratio were also studied, which presented a reduction in O/C and H/C molar ratio. The expected decline in O/C molar ratio was primarily due to the dehydration by thermal treatment of BM, which consequently resulted in decreased polarity and hydrophilicity in BC. However, the decline in H/C molar ratio might be due to the higher carbonization and aromaticity of the resulting BC [35,36,37]. The lower (O + N)/C ratio can be explained by the presence of a low number of polar surface functional groups and higher aromaticity of the produced BC [38,39].

**Figure 1:**
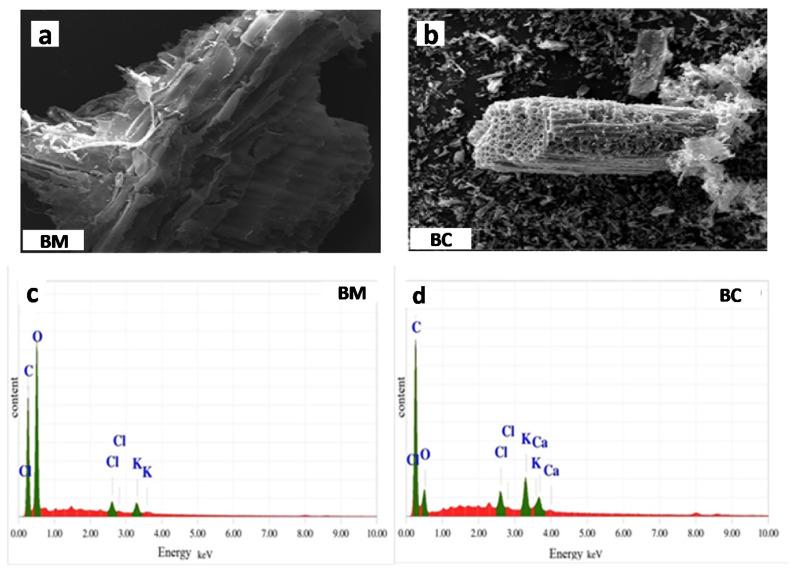
Scanning electron microscopy images of the (**a**) mesquite wood (BM) and (**b**) BM-derived biochar (BC), as well as energy-dispersive X-ray spectroscopy (EDS) analyses of (**c**) BM and (**d**) BC.

**Figure 2:**
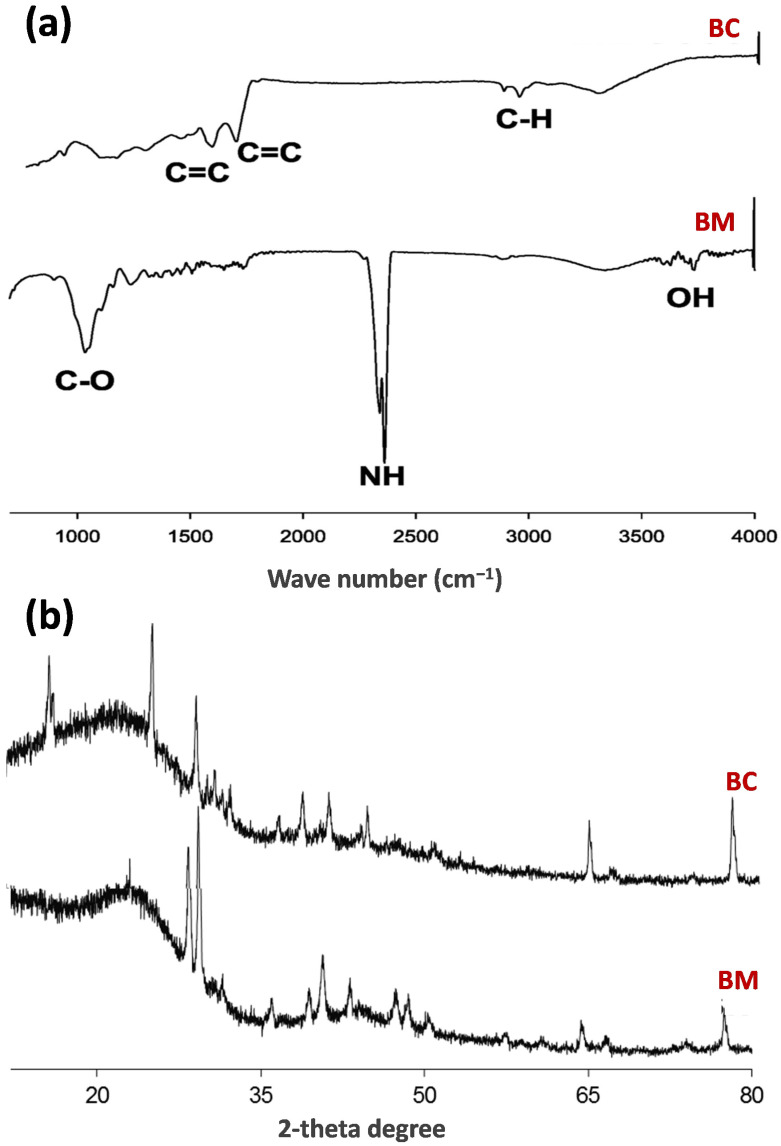
Fourier-transform infrared spectroscopy patterns (**a**) and X-ray diffraction analyses (**b**) of mesquite wood (BM) and BM-derived biochar (BC).

SEM analysis of the materials showed a more porous structure in BC than that of raw BM (Figure 1a,b). The oval-shaped porous structure of BC could be the result of loss of organic compounds and volatiles due to higher carbonization temperature [38,40]. On the other hand, energy-dispersive X-ray spectroscopy analysis described O and C as dominant elements in BM, followed by Cl (Figure 1c,d). After pyrolysis treatment, a sharp decline in O contents was observed in BC, while C elements were further concentrated. Additionally, along with minor Cl and K, Ca was also found in BC as a minor element. The FTIR spectra of BM showed small OH stretching around 3600 cm^−1^, which indicated the presence of a hydroxyl group associated with water molecules (Figure 2a). Moreover, bands at 2350 cm^−1^ in BM indicated the presence of amine (NH) functional groups, which were not found in BC. Likewise, C–O bands at 1025 cm^−1^ were attributed to the presence of cellulose, lignin, and hemicellulose in BM, which disappeared in BC. Due to the thermalization process, newly detected bands in BC at 2940 cm^−1^ are ascribed to C–H aromatic stretching. Likewise, new bands of C=C appeared in BC at 1560 and 1600 cm^−1^ as a result of the thermal process. Overall, FTIR spectra showed the presence of organic and aromatic compounds in raw BM and BC, respectively [41]. The mineral composition of the BM and BC as analyzed by XRD is shown in Figure 2b. Peaks of organic compounds (cellulose) and whewellite (CaC_2_O_4_.H_2_O) were detected in BM, which laterally disappeared in BC after thermalization. A similar trend was reported by Al-Wabel et al. [35], stating that the cellulose and calcium oxalate peaks in raw BM disappeared when it was pyrolyzed at 400 °C or above. Peaks appearing at 3.14, 2.24, 2.05, and 1.44 Å were designated to the presence of CaCO_3_. Likewise, the peaks appearing at 2.81, 2.28, and 1.86 Å were ascribed to calcium hydroxyapatite [Ca(PO_4_)_3_(OH)]. Therefore, these results revealed that the pyrolysis process induced substantial changes in the proximate, elemental, structural, and mineral composition of BC. 

### 2.3. Effects of Soil Type on Doxycycline Sorption 

Owing to the application of animal waste, doxycycline (DC) has frequently been detected in the environment, especially in soil. Soil clay contents, organic matter, pH, and metal oxides are the important factors responsible for DC retention in soil [42]. In this context, the DC sorption behavior in soil varies with soil type due to the varying physiochemical properties of soil. Therefore, three soils with contrasting characteristics were selected to investigate their DC retention capacity in this study. Soils S1, S2, and S3 were mixed with manure to produce MS1, MS2, and MS3, which were further amended with BC to produce BMS1, BMS2, and BMS3. Pearson correlations of DC sorption onto soil alone and in combination with manure and/or BC with physiochemical characteristics of soils are shown in Table 3. Soil pH exhibited significant negative correlations (*p* < 0.05) with DC sorption onto soil alone and in combination with manure and/or BC. Significant positive correlations (*p* < 0.05) of DC sorption with soil organic matter and clay contents were seen in soil alone and when combined with BC and manure, while soil P concentration positively correlated (*p* < 0.05) with DC sorption onto the soil + manure system only. The positive correlation of DC sorption with clay could be explained with ion exchange between amine groups of DC and surfaces of clay minerals. Similar findings have already been reported by Wang et al. [43]. Likewise, in another study, Aristilde et al. [44] reported that structural charge localization as well as interlayer spacing of clay minerals in soil facilitated antibiotic binding, which subsequently resulted in multiple sorption processes. Kim et al. [45] studied the sorption characteristics of various antibiotics in soil and concluded that clay minerals, pH, organic matter, and texture were the major factors responsible for antibiotic sorption and retention in soil. Thus, depending on the physiochemical characteristics of soil, the behavior of antibiotic retention, sorption, leaching, and dissipation varies with soil types [45]. The positive correlation of DC sorption with organic matter could be due to binding of DC molecules with aromatic organic substances via H bonding and van der Waals forces. Moreover, Kahle and Stamm [46] reported that the amine groups of DC may interact with alcoholic, carboxylic, enol-, and phenolic groups of organic matter, subsequently improving DC retention in soils. 

**Figure 3:**
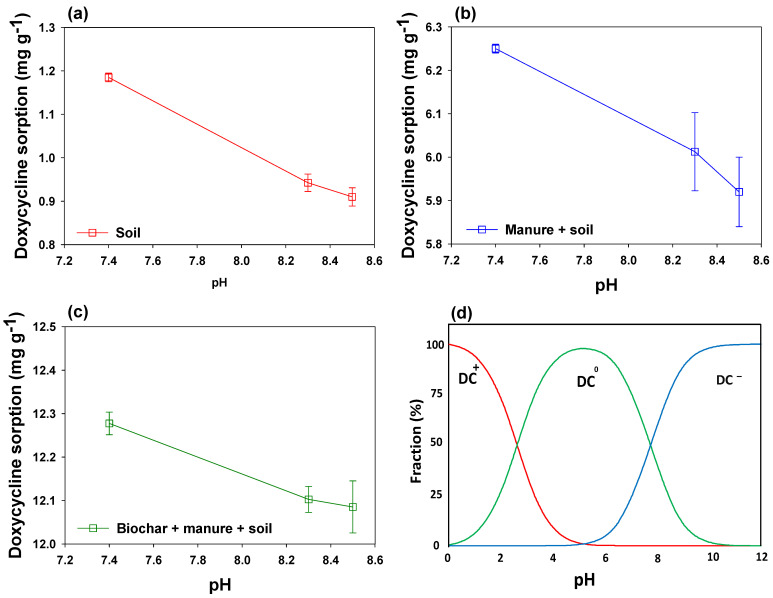
Effect of soil pH on doxycycline sorption onto (**a**) soil, (**b**) soil + manure, and (**c**) soil + manure + mesquite wood-derived biochar, as well as (**d**) transformation of DC forms with changing pH.

Among soil properties affecting DC sorption, pH is a key factor, as it affects the surface charges of the adsorptive materials and species distribution of DC. Thus, to understand the effects of pH on DC sorption, three soils with different pH values were selected for this study. A stronger negative relation of soil pH and DC sorption was observed in this study. To further explore the interactions of pH and DC sorption, the changes in DC sorption and speciation with pH changes are shown in Figure 3. Soil alone with a pH of 7.41 showed the highest DC sorption (1.185 mg g^−1^), which declined by 27.7% and 30.2% with a rise in pH to 8.32 and 8.48, respectively (Figure 3a–c). However, the decrease in DC sorption with the rise in pH from 7.41 to 8.32 and 8.48 was in the range of 1.5% to 5.6%. The decline in DC sorption with increasing soil pH could be attributed to the changes in soil physicochemical characteristics and variations in electrostatic forces as a result of changes in speciation of DC at different pH levels (Figure 3d). DC molecule is amphoteric in nature, which can act as a cation, a zwitterion, or as an anion depending upon the pH of the media. Various ionizable functional groups, including alcohol, amino groups, and phenols, are possessed by DC molecules at different pH levels [47]. Zhang et al. [47] reported that a DC molecule acts as a cation (DC^+^) at pH below 3.5, a zwitterion (DC^0^) in the pH range of 3.5–7.7, and as an anion (DC^−^) in the pH range of 7.7 to 9.5. As the pH of soil S1 was 7.41 in the current study, the DC molecules acted as zwitterions, whereas they acted as anions in soil S2 and S3 due to their pH of 8.32 and 8.48, respectively. Therefore, lower DC sorption at pH 8.32 and 8.48 could be due to electrostatic repulsive forces between DC and soil/manure/biochar particles. 

The higher DC sorption at pH 7.41 as compared to pH 8.32 and pH 8.48 depicted the involvement of other mechanisms in DC sorption too. Non-electrostatic interactions between BC/manure particles and DC might have contributed to DC sorption as well [48]. The higher DC sorption at pH 7.41 onto soil alone, as well as amended with manure and BC, could be due to the development of π–π electron–donor–acceptor (EDA) interactions and H bonding between DC zwitterions and organic components of the adsorbents, and this is why soil S1 with manure and BC resulted in higher DC sorption than S1 alone. Previously, Wei et al. [49] has reported the involvement of π–π EDA interactions and H bonding for the adsorption of DC onto Fe-loaded sludge-derived biochar. However, with an increase in pH, the π-withdrawing capability of DC decreases substantially, which hinders the further sorption of DC [50]. Nevertheless, DC sorption onto the materials, even at pH 8.32 and 8.48, despite electrostatic repulsion, could be attributed to H bonding interactions [51]. Furthermore, deprotonation of oxygen-containing functional groups (O–H and C–O) of biochar at pH 8.32 and 8.48 might have resulted in the development of negative charges on the surface of BC, subsequently generating electrostatic repulsion between BC and DC [52]. A negative correlation between the concentration of tetracycline antibiotics in manure and pH has already been reported by Conde-Cid et al. [53], indicating their lower adsorption onto manure at higher pH levels. Likewise, Park and Huwe [54] found reduced tetracycline sorption onto manure with increasing pH of manure. Similarly, Al-Wabel et al. [55] observed higher leaching of sulfamethoxazole at higher pH levels. Thus, the protonation of DC at higher pH developed anionic DC species, which were repelled by the negatively charged organic matter present in the manure, consequently decreasing the sorption of DC onto manure [36]. Therefore, the complexation of DC with organic matter could reduce its bioavailability to the bacteria and plants, consequently avoiding the risk of antibiotic resistance generation [56]. Besides, physiochemical properties of soils, such as pH, CEC, and clay contents, as well the presence of water, substantially affect the bioavailability of DC in different soils [57]. Pan and Chu [58] reported that TC molecules possess a higher half-life and stronger sorption affinity towards soils. Thus, a longer contact time results in the development of strong sorption as well as diffusion of DC molecules into soil pores, subsequently reducing its bioavailability to the micro-organisms [58]. Therefore, soil S1 along with manure resulted in higher DC sorption as compared to S2 and S3 with manure, which was due to the lower pH of S1 than that of S2 and S3. These results suggested that the soils with neutral pH can potentially sorb higher amounts of DC, whereas the soils having a pH of >7.7 possess lower sorption capacity and higher mobility for DC antibiotics.

### 2.4. Sorption Kinetics 

**Figure 4:**
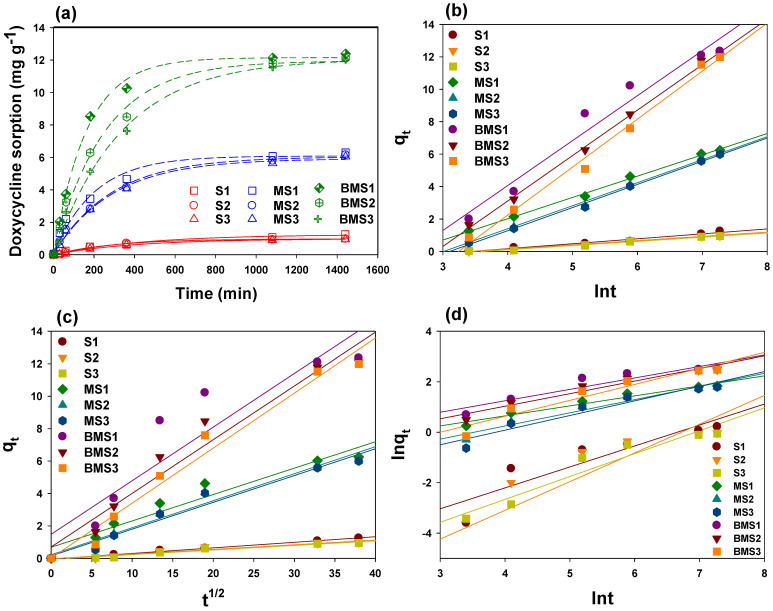
(**a**) Doxycycline sorption kinetics onto different soils (pH 7.41 (S1), pH 8.32 (S2), and pH 8.48 (S3)), manure + S1 (MS1), manure + S2 (MS2), manure + S3 (MS3), mesquite wood-waste-derived biochar + MS1 (BMS1), mesquite wood-waste-derived biochar + MS2 (BMS2), and mesquite wood-waste-derived biochar + MS3 (BMS3), as well as Elovich (**b**), intraparticle diffusion (**c**), and power function (**d**) plots of kinetics model fittings.

Sorption kinetics trials were conducted at a constant temperature to study the dynamics of DC sorption onto the soil with and without manure and BC application. The effects of contact time on DC sorption are presented in Figure 4a. Overall, the highest DC sorption was demonstrated by soil combined with manure and biochar, followed by soil amended with manure, whereas only soil exhibited the lowest DC sorption. The dynamics of DC sorption can be divided into three discrete phases, i.e., an initial rapid phase (0–200 min), a relatively slower phase (200–400 min), and an equilibrium stage. The first sorption phase in soil with BC and manure was quicker as compared to only soil and soil with manure. Frequent DC sorption at the beginning could be attributed to the presence of more active sorption sites, which were occupied with time [49]. To further understand the sorption process, kinetics models were employed to the data. The estimated parameters of the kinetic models, along with *R*^2^ values, are presented in Table 4. The calculated *R*^2^ values indicated that DC sorption data was best described by Elovich kinetic model (*R*^2^ = 0.965–0.997), followed by intraparticle diffusion (*R*^2^ = 0.871–0.984) and power function (*R*^2^ = 0.751–0.962). The plots of best fitted kinetic models, i.e., Elovich, intraparticle diffusion, and power function, are shown in Figure 4b–d, respectively. The suitability of Elovich and power function kinetic models depicted chemical sorption of DC onto the adsorptive materials used in this study, while the fitness of the intraparticle diffusion suggested the diffusion into the pores.

The parameters derived from the simulations of the kinetic models are shown in Table 4. The Elovich-model-predicted sorption constant (β) values were lower for DC sorption to S1, S2, and S3 (0.243–0.251), while the highest for BMS1, BMS2, and BMS3 (1.914–2.611). Similarly, the intraparticle-diffusion-model-predicted constant (c) and Elovich-model-predicted initial sorption constants (α) were highest in BMS1, BMS2, and BMS3. Specifically, the highest c and α were generated by BMS1 (1.897 and 1.485 mg g^−1^ min^−1^, respectively), followed by BMS2 (1.793 and 0.698 mg g^−1^ min^−1^, respectively), suggesting quicker sorption of DC onto soils S1 and S2 combined with manure and BC. Similarly, diffusion rate constant (k_id_) values were higher for soils with manure and BC as compared to soil with manure or soil alone. The highest k_id_ value for BMS3 (0.339 (mg g^−1^)^−0.5^), followed by BMS2 (0.331 (mg g^−1^)^−0.5)^ and BMS1 (0.330 (mg g^−1^)^−0.5^), depicted a higher diffusion of DC into the pores of these materials, probably owing to the presence of BC, which is a highly porous material. The DC molecules might have been transported from the aqueous phase to the surface of sorptive materials, and then diffused into the pore. Previously, Wei et al. [49] reported the diffusion of DC and TC molecules into the pores of iron-loaded sludge-derived biochar. Similarly, power-function-model-predicted rate coefficient (k_f_) values were higher for BMS1 (0.383 mg g^−1^ min^−1^), BMS2 (0.378 mg g^−1^ min^−1^), and BMS3 (0.396 mg g^−1^ min^−1^). Therefore, the best fitness of the Elovich, power function and intraparticle diffusion model suggested multiple mechanisms for DC sorption onto the tested adsorptive materials, including chemical sorption and pore diffusion. As the sorption process is mainly influenced by physiochemical properties of both adsorbent and adsorbate, as well as the prevailing conditions of the media, DC sorption mechanisms differed with varying the type of soil [59]. The involvement of various soil characteristics, including clay minerals, pH, organic matter, and soil texture, in the retention of DC onto adsorptive materials has previously been reported by Kim et al. [45]. Among these factors, pH is of crucial importance due to the amphoteric nature of DC molecules. Therefore, soil S1 amended with manure and/or BC exhibited relatively higher DC retention due to lower pH as compared to S2 and S3. The higher sorption of DC onto soil S1 amended with BC could be due to the development of π–π EDA interactions and H bonding due to the existence of DC zwitterion at pH 7.41 [49,50,51]. However, in sorptive materials having soil S2 and S3, the DC molecules acted as anions and, hence, electrostatic repulsive forces resulted in decreased DC sorption [52].

### 2.5. Sorption Equilibrium 

**Figure 5:**
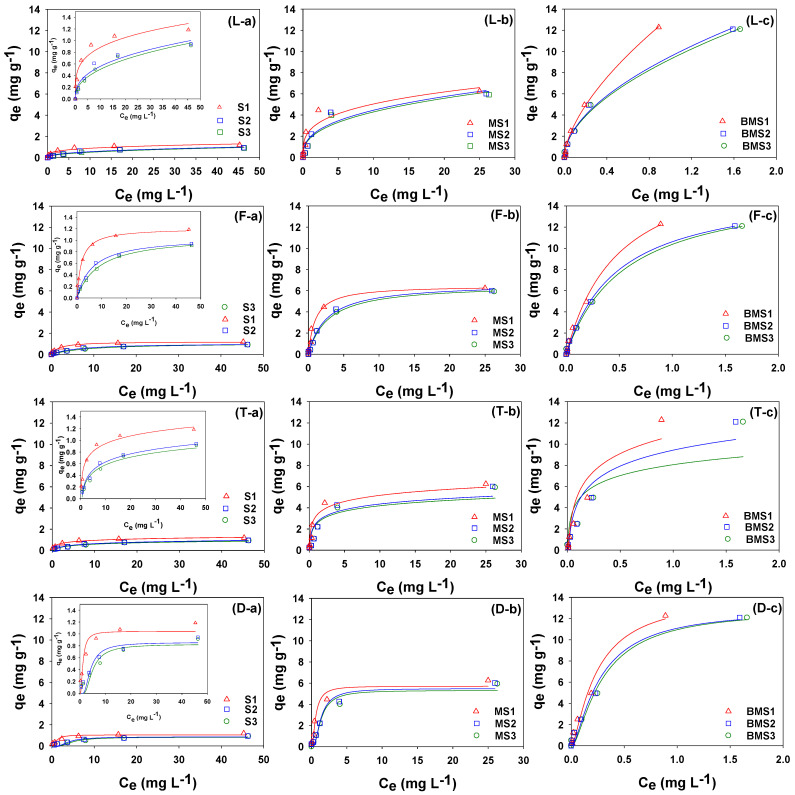
Doxycycline sorption fittings on Langmuir (**L-a**, **L-b**, and **L-c**), Freundlich (**F-a**, **F-b**, and **F-c**), Temkin (**T-a**, **T-b**, and **T-c**), and Dubinin–Radushkevich (**D-a**, **D-b**, and **D-c**) isotherm models by different soils (pH 7.41 (S1), pH 8.32 (S2), and pH 8.48 (S3)), manure + S1 (MS1), manure + S2 (MS2), manure + S3 (MS3), mesquite wood-waste-derived biochar + MS1 (BMS1), mesquite wood-waste-derived biochar + + MS2 (BMS2), and mesquite wood-waste-derived biochar + MS3 (BMS3).

The tendency of DC sorption onto soil amended with manure and BC was investigated in equilibrium sorption batch trials at a constant temperature and sorbent dose, but different initial DC concentrations. Nonlinear forms of various isotherm models were employed to the DC sorption data to study sorption mechanism. The isotherm curves generated by simulating the DC sorption data are presented in Figure 5. Overall, DC sorption onto all the tested adsorptive materials decreased with increasing initial DC concentrations, which could be owing to the decrease in adsorptive site abundance with increasing DC concentration [27,60]. This can also be evidenced with an H-type (high affinity) isotherm generation at a lower initial DC concentration, indicating an abundance of active sorption sites. Nevertheless, increasing the initial DC concentrations results in changing the isotherm shape into L-type (low affinity), indicating a lower availability of active sorption sites. 

The estimated parameters of nonlinear isotherm models, including Langmuir, Freundlich, Temkin, and Dubinin-Radushkevich are presented in Table 5. Based on *R*^2^ values, it was seen that the DC sorption data were best described by Langmuir isotherm (*R*^2^ = 0.984–0.999), followed by Freundlich (*R*^2^ = 0.916–0.996), Dubinin-Radushkevich (*R*^2^ = 0.863–0.984), and Temkin (*R*^2^ = 0.725–0.980). Langmuir model predicted maximum DC sorption capacity (Q_L_) was exhibited by BMS1 (18.930 mg g^−1^), followed by BMS3 (16.073 mg g^−1^) and BMS2 (15.792 mg g^−1^). Similarly, Dubinin-Radushkevich predicted sorption capacity (q_D_) was the highest for BMS1 (13.833 mg g^−1^), followed by BMS3 (12.777 mg g^−1^) and BMS2 (12.765 mg g^−1^). The DC sorption capacities of the tested adsorptive materials followed the order of: BMS1 > BMS3 > BMS2 > MS1 MS2 ≥ MS3 > S1 > S2 > S3. Overall, the DC sorption capacity of soil increased by 5–6.5-fold with the addition of manure into the soil, whereas it increased up to 10–13 times with addition of both manure and BC. Further, DC sorption capacity of soil amended with manure and BC was double the sorption capacity of soil with only manure. This gives a clear indication of the higher DC sorption capacity of soil having organic components within it, such as manure and BC. These results are also in line with the results of kinetic modeling simulations, where an increased sorption rate was observed with the addition of biochar and manure into the soil. Akin to this, BMS1, BMS2, and BMS3 showed higher values of Freundlich-model-predicted K_F_ (13.263, 9.518, and 9.266 L g^−1^, respectively). Moreover, Freundlich-model-predicted 1/*n* values for all the sorptive materials were below unity (0.0253–0.618), suggesting suitability of DC sorption onto soil with and without manure/BC amendments [60].

The fitness of Langmuir and Freundlich isotherm models to sorption data suggested that DC sorption onto the tested materials followed both monolayer and multilayer. However, Dubinin-Radushkevich-predicted E values were <8.0 kJ g^−1^ (1.0 × 10^−4^–9.1 × 10^−3^), which indicated that the sorption of DC did not follow an ion-exchange mechanism [60]. Therefore, the suitability of Langmuir, Freundlich, Temkin, and Dubinin-Radushkevich isotherm models depicted the involvement of multiple mechanisms in the sorption of DC in soil with and without the addition of manure and BC. The higher sorption of DC onto soil S1 alone or in combination with manure and/or BC could be due to lower pH (7.41) of S1 as comparted to S2 and S3. At this pH, DC molecules were prevailing as neutral ions (DC^0^), which might have developed π–π EDA and H bonds with organic components of the sorptive materials, consequently resulting in higher DC sorption [50,51]. However, in soil S2 and S3, due to higher pH levels (8.32 and 8.48, respectively), DC molecules were transformed into anions, which were subsequently repelled by the negatively charged surfaces of either soil, manure, or BC. Previously, Sassman and Lee [61] reported similar results, claiming that TC sorption onto soil decreased at higher pH due to electrostatic interactions. Similar findings were also reported by Tan et al. [52]. The significantly higher DC sorption of soil amended with BC could be due to higher surface charge, functional groups, and porosity of the BC materials which have developed π–π EDA and H bonds [49,50,51]. Therefore, the addition of BC into DC-laden manure-amended soil could be an effective and cost-efficient strategy to decrease DC mobility and mitigate its toxicity. 

### 2.6. Desorption of Doxycycline

**Figure 6:**
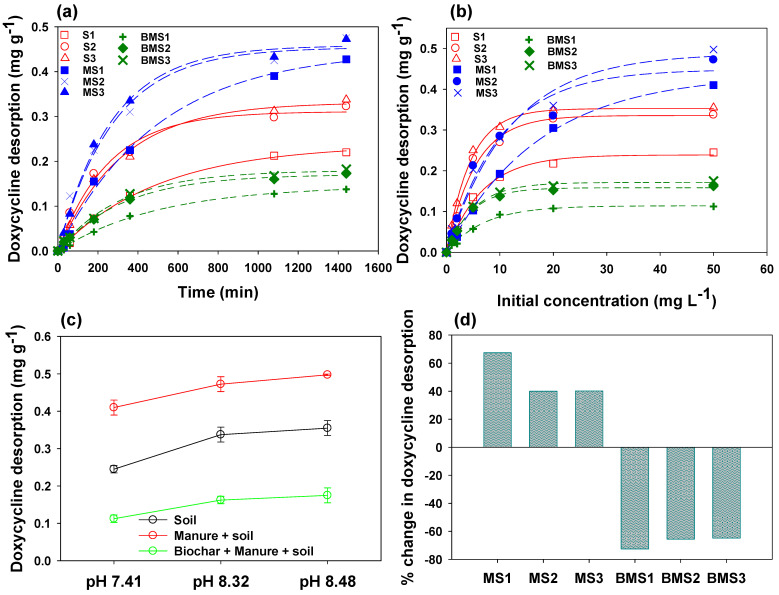
Effects of contact time (**a**), initial concentration (**b**), and soil pH (**c**) on doxycycline desorption from different soils (pH 7.41 (S1), pH 8.32 (S2), and pH 8.48 (S3)), manure + S1 (MS1), manure + S2 (MS2), manure + S3 (MS3), mesquite wood-waste-derived biochar + MS1 (BMS1), mesquite wood-waste-derived biochar + MS2 (BMS2), and mesquite wood-waste-derived biochar + MS3 (BMS3), as well as percent increase/decrease in doxycycline desorption from soils S1, S2, and S3 after the addition of manure and manure + mesquite wood-waste-derived biochar (**d**).

Desorption is a critically important process to monitor the bioavailability and transport of DC in the environment, especially in soil. Thus, desorption behavior of DC from loaded soils with and without manure and BC were investigated, and results are presented in Figure 6. The effect of contact time on DC desorption is shown in Figure 6a, which indicated three distinct phases, i.e., an initial rapid desorption stage (0–230 min), a relatively lower desorption stage (230–500 min), and equilibrium. Overall, the higher DC desorbed rate was observed in soils amended with manure, followed by soil alone and soils amended with manure and BC. This shows that the soils amended with BC were slower in desorbing the DC as compared to the soil alone and soil amended with manure. Further, the desorption of DC is significantly influenced by the initial concentration of DC, as shown in Figure 6b. The desorption of DC increased with an increasing initial DC concentration in all the tested materials. The desorption was higher with lower initial DC concentration, which slowed down gradually with an increase in DC concentration. At lower initial concentrations, S1, S2, and S3 exhibited the higher desorption; however, at higher initial DC concentrations, the highest DC desorption was shown by MS1, MS2, and MS3, while the lowest desorption was shown by BMS1, BMS2, and BMS3. Overall, the order of desorption was: MS3 > MS2 > MS1 > S3 > S2 > S1 > BMS3 > BMS2 > BMS1. Therefore, these results suggested that BMS1, BMS2, and BMS3 were the most efficient adsorbent for DC, as they exhibited higher DC sorption and lower desorption, thus depicting the significance of BC in reducing DC mobility in soil. However, the physiochemical properties of the soil affect the mobility of DC in soil as well. Therefore, the impacts of soil pH on DC desorption were also investigated in this study, as shown in Figure 6c. Significantly lower desorption of DC was seen in soil with a pH of 7.41, which increased in soils with pH of 8.32 and 8.48. The results indicated that DC desorption increased by 38% and 45% in S2 and S3, respectively, as compared to S1. Likewise, the rise in desorption reached up to 15% and 21% in MS2 and MS3, respectively, as compared to MS1, and up to 44% and 56% in BMS2 and BMS3, respectively, as compared to BMS1. These variations in DC desorption due to the changes in soil pH were owing to the amphoteric nature of DC molecule [62]. In the current study, DC was present as zwitterions in S1-based sorptive materials, and as anions in S2- and S3-based materials. Thus, the higher desorption in S1 and S2 alone or in combination with manure and BC was due to electrostatic repulsive forces. 

The significance of BC in reducing DC transportation and enhancing its retention in the soil is presented in Figure 6d. The desorption of DC was increased by 67%, 40%, and 41% in MS1, MS2, and MS3, as compared to S1, S2, and S3, respectively, indicating potentially higher mobility of DC in the soil after manure application. Contrarily, the addition of BC to soil has significantly reduced the desorption of DC. It can be observed that the DC desorption was deceased by 73%, 66%, and 65% in BMS1, BMS2, and BMS3, as compared to S1, S2, and S3, respectively. Previous studies demonstrated that the occurrence of ample functional groups, porous structure, higher functionality, surface charges, and aromaticity make the BC an efficient sorbent for organic contaminants, such as antibiotics [52,63]. The graphitized structure of BC can develop π–π EDA interactions with organic compounds, which, in turn, result in higher sorption of the contaminants onto BC surface [64]. Moreover, the O-containing surface functional groups, such as alkoxy, carboxyl, and hydroxyl, can potentially generate H bonds with phenolic hydroxyl or amino functional groups of DC, subsequently enhancing its sorption onto BC [49,50,51,57]. Besides, Abel et al. [65] found that DC molecules can be captured by the external surfaces of the BC particles, which are then transported into the pores and captured into internal surfaces of BC. Therefore, these multiple mechanisms have resulted in stronger sorption of DC onto BC-amended soil, consequently desorbing lower amounts of DC back into the system. Therefore, the application of BC to the contaminated soil could potentially reduce the mobility of DC in soil and enhance its retention, subsequently reducing the environmental pollution. 

### 2.7. Environmental Implications 

Toxicokinetic studies revealed that approximately 90–95% of DC is not metabolized in an animal’s body and is released into the environment with manure and urine [66]. Application of this DC-laden manure into soil results in the release of DC into the environment. Leaching, run-off, drainage, and transmission of residual DC result in underground and surface water contamination and soil pollution [67]. Further, plants can uptake these residues, and consequently add them into the food-chain. Therefore, retention and immobilization of DC in soil after manure application must be enhanced to avoid environmental contamination. However, the removal and sorption of DC is a complicated process due to its amphoteric nature. The results of the current study demonstrated the significance of BC in enhancing the retention and immobilization of DC in a soil–manure system. It was observed that the sorption of DC in soils with a pH of >7.7 was lower, suggesting that alkaline soils had a higher potential for DC leaching into deeper soil layers. Contrarily, soil with a pH of 7.41 demonstrated higher DC sorption and lower desorption, indicating its higher DC retention capacity. Thus, information about soil properties, such as pH, organic matter, texture, and clay minerals, should be collected before planning the remediation of a specific soil contaminated with antibiotics. However, application of BC resulted in a significant reduction in DC desorption, even in alkaline soils. For instance, DC desorption was reduced by 66% and 65% with the application of BC to S2 and S3. Therefore, BC application can be used as an efficient and low-cost strategy to enhance DC retention and limit its mobility in contaminated soils. As BC is carbonaceous material with higher porosity, large surface area, and ample functional groups, its application to the contaminated soil may result in immobilization of DC and other contaminants, improved physiochemical properties of soil, as well as improved nutrient and water holding capacity. Moreover, as BC is produced by pyrolyzing the waste materials, it can add a lot of economic benefits as well. Nevertheless, further investigations are needed to evaluate the performance, ecotoxicological effects, economic benefits, and environmental implications of BC under real soil systems. Therefore, findings of this study are of significant importance in mitigating the hazardous effects of DC toxicity in the environment.

## 3. Materials and Methods

### 3.1. Materials

Doxycycline was purchased from Sigma–Aldrich Inc., St. Louis, MO, USA, and oxalic acid, Na_2_HPO_4_, citric acid, and Na_2_EDTA were acquired from Fisher Scientific Co. (Springfield, NJ, USA). HPLC grade methanol and acetonitrile were purchased from Merck (Darmstadt, Germany). Strata™ X SPE cartridge was acquired from Phenomenex, Torrance, CA, USA. De-ionized water was produced through Milli-Q, Germany, with 18.2 MΩ cm^−1^ resistivity.

### 3.2. Soil and Manure Sample Collection and Characterization

Soil and manure samples were collected from Al-Kharj city (24° 11.995′ N, 47° 25.386′ E and 24° 14.481′ N, 047° 29.886′ E, respectively), Saudi Arabia, which is recognized for its dairy products and agricultural practices. A composite manure sample was collected in an acetone-rinsed plastic container from a dairy farm and stored in an ice-box. Composite soil samples were collected from a depth of 0–30 cm using a soil auger from different locations. The collected soil and manure samples were transferred to the laboratory for further analysis. Sub-samples of soil and manure were taken, dried in air, grinded, and passed through a 2-mm screen for further analyses. The collected samples were analyzed for moisture, EC, pH, organic matter, CEC, CaCO_3_, and available P, K, and Na by following the standard procedures. EC and pH were determined in 1.25 ratio suspension in deionized water. Organic matter was analyzed by Walkley–Black [68]. Available P was extracted with AB-DTPA and analyzed with a UV/Vis spectrophotometer (Lambda EZ 150, PerkinElmer, Norwalk, CT, USA). Malvern Mastersizer 2000 was used to investigate the particle size distribution of the collected soil to determine the texture. Moreover, the manure sample was subjected to proximate analyses to determine moisture, ash, volatiles, and fixed carbon contents [69]. To measure the concentration of DC in soil and manure samples, the McIlvain extraction method was employed, as elaborated by Al-Wabel et al. [70]. Briefly, 20 mL of McIlvain buffer (625 mL of 0.2 M Na_2_HPO_4_ and 1000 mL of 0.1 M citric acid) and 200 µL of 5% Na_2_EDTA were mixed with 1 g of samples. After shaking for 20 min at 200 rpm, the suspension was centrifuged and supernatant was collected. A total of three extracts were collected from the same sample and subjected to a clean-up process.

### 3.3. Biochar Production and Characterization

Mesquite wood waste was collected, placed in an oven at 60 °C for drying, ground, and sieved to obtain a fine particle size. The ground mesquite wood waste biomass (BM) was pyrolyzed using a muffle furnace (WiseTherm; Daihan Scientific, Seoul, South Korea) at 600 °C for a time period of 240 min in an air-tight sealed container made of stainless steel (height: 22 cm and diameter: 7 cm) [31]. The prepared mesquite biochar (BC) was ground after cooling to room temperature, sieved through a 2-mm screen, and stored for further analyses. To investigate the contents of moisture, volatiles, ash, and fixed carbon, BC was subjected to proximate analyses [69]. Biochar was also subjected to McIlvain buffer extraction, as described in the previous section for DC analysis [70]. CHNS-O elemental analyzer (PerkinElmer series II, Waltham, MA, USA) was used to determine the contents of C, H, N, and S in the synthesized BC, while the contents of O were determined through the difference method. Molar ratios of O:C and H:C were also calculated in the synthesized biochar samples based on the elemental composition. The Brunauer–Emmett–Teller (BET) surface area of BC was investigated using an ASAP-2020 surface area and microporosity analyzer (Micromeritics, Norcross, GA, USA). The surface structure of the prepared BC was analyzed using inspect S-50 scanning electron microscope (SEM, FEI, The Netherlands). The crystallographic structure of the prepared BC was investigated by the XRD-7000 (Shimadzu Corp, Kyoto, Japan), whereas Fourier-transform infrared spectroscopy (Nicolet 6700 FTIR: Thermo Scientific, Waltham, MA, USA) was used to identify the functional groups of the prepared BC.

### 3.4. Sorption–Desorption Experiments

**Scheme 1:**
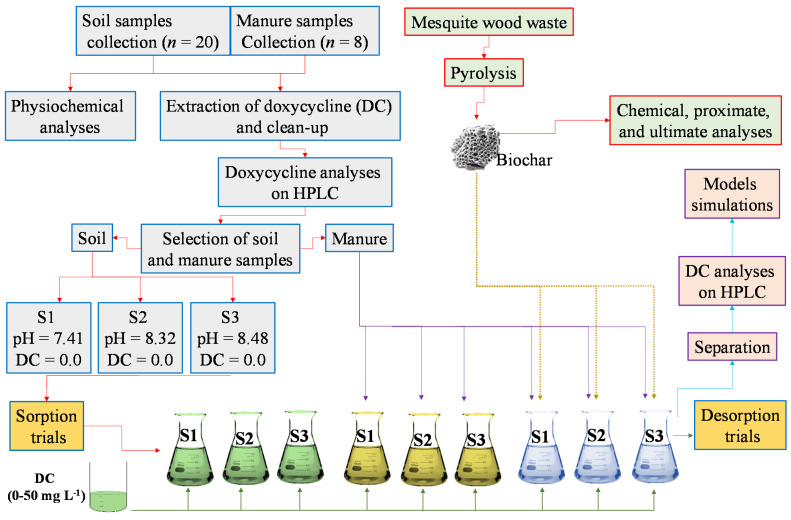
Schematic representation for soil and manure sample collection, characterization, and doxycyline sorption–desorption batch set-up.

Near neutral to alkaline pH is the most commonly prevailing soil pH in the study area. Therefore, the soils with pH of 7.41, pH 8.32, and pH 8.48 were selected from the collected and analyzed soil samples for sorption–desorption studies. The selected soils with contrasting physiochemical properties, i.e., soil S1 (pH 7.41), S2 (pH 8.32), and S3 (pH 8.48) were mixed with grinded manure at the rate of 5.0% (*w*/*w* ratio) and labeled as MS1, MS2, and MS3, respectively. Further, MS1, MS2, and MS3 were amended with 2.0% (*w*/*w* ratio) of BC and named as BMS1, BMS2, and BMS3. These sorptive materials, i.e., only soils, manure + soil, and BC + manure + soil, were tested for their DC sorption capacity via sorption batch trials. All the trials were performed at room temperature, i.e., 23 ± 2 °C. A total of 2.0 g of dried sorptive material (either only soils, manure + soil, or BC + manure + soil) was suspended in 50 mL of 0.01 M CaCl_2_ (background solution) containing a specific concentration of DC in all sorption trials. Additionally, 0.02% of sodium azide was added to limit bacterial activities. The suspension was thereafter covered properly to stop photodegradation of DC and shaken on a mechanical shaker at 180 rpm for a specific time interval at room temperature. For kinetic sorption experiments, an initial DC concentration of 50 mg L^−1^ was used and the samples were withdrawn from the shaker after 0.0, 30, 60, 180, 360, 1080, and 1440 min. For equilibrium sorption trials, various initial DC concentrations (0.0, 1.0, 2.0, 5.0, 10, 20, and 50 mg L^−1^) were used and the samples were withdrawn from the shaker after 1440 min. Thereafter, the solution was separated from the suspension through centrifugation for 20 min at 3500 rpm and subjected for determining the remaining DC concentration in solution. The residual solids after removing the supernatant solution were used for desorption experiments using freshly prepared 0.01 M CaCl_2_ solution with 0.02% of sodium azide. For the desorption kinetics experiments, 50 mg L^−1^ of DC was added into the remaining solids, and the suspension was shaken for 0.0–1440 min, whereas, for equilibrium sorption trials, 0.0–50 mg L^−1^ of DC was used and the suspension was shaken for 1440 min. Then, solution was separated through centrifugation and subjected to DC analyses. Scheme 1 represents the soil and manure sampling, characterization, and DC sorption–desorption batch set-up. 

### 3.5. Solid Phase Extraction 

Solid phase extraction (SPE) was performed with the help the HLB cartridges (3 mL/60 mg) for clean-up and concentration. HLB cartridges were initially activated by using 3.0 mL methanol, 3.0 mL 0.5 N HCl, and 3.0 mL of water (HPLC grade). Then, the extracts were passed through these cartridges using a pressure of 40 psi of vacuum and a flow rate of ~2 mL min^−1^. When all the extracts had been passed, the cartridges were washed with 3 mL HPLC-grade water and were set for drying in vacuum for 30 min. Thereafter, 2.5 mL of acetonitrile was used for the elution of DC, the eluted DC was collected in the collection vials and mixed using a vortex mixer. From the collection vials, acetonitrile was evaporated by heating at 30 °C in the presence of N_2_ atmosphere. Then, it was reconstituted in the original background solution, filtered with 0.45 µm syringe filters, and poured into HPLC vials for analyses [71].

### 3.6. Quantification of Doxycycline

Prepared and cleaned-up extracts were used to analyze the concentrations of DC using high-performance liquid chromatography (HPLC; Prominence-i, LC-2030C, Shimadzu, Kyoto, Japan), with a reversed-phase Raptor C18 column (100 mm × 21 mm, 2.7 μm particle size) and PDA detector. Three mobile phases, i.e., methanol (A), acetonitrile (B), and 0.1 M oxalic acid (C) with pH adjusted at 3.0 were used for DC quantification. Analyses were performed in a gradient system with a mobile phase composition of 7:8:85 (A:B:C) for 0.0–15 min, then 10:20:70 (A:B:C) for 15–20 min, with a flow rate of 0.5 mL min^−1^, λ of 354 nm, and column temperature of 35 °C. Using DC concentration in standards versus absorbance (%) by HPLC, a calibration curve (*R*^2^ ~ 0.99) was constructed. The standards were also run as samples of unknown concentrations for quality assurance. 

### 3.7. Kinetics Modeling 

The amount of DC sorbed onto the sorptive material was calculated using Equation (1):

(1) $$q_{e}=\left[\frac{C_{o}-C_{e}}{m}\right]\times v$$

where *C_o_* represents initial concentrations (mg L^−1^), *C_e_* represents DC at equilibrium (mg L^−1^), *q_e_* is the amount of DC sorbed (mg g^−1^), *m* represents the weight of adsorptive material (g), and *v* the used volume in L.

Kinetics models were used to explore the dynamics of DC sorption, as shown in Equations (2)–(7) [72,73]:

(2) $$\mathrm{First}-\mathrm{order}\;lnq_{t}=lnq_{o}-k_{1}t$$

(3) $$\mathrm{Second}-\mathrm{order}\;\frac{1}{q_{t}}=\frac{1}{q_{o}}-k_{2}t$$

(4) $$\mathrm{Pseudo}-\mathrm{second}-\mathrm{order}\;\frac{t}{q_{t}}=\frac{1}{k_{2}^{\prime }q_{e}^{2}}+\frac{1}{q_{e}}t$$

(5) $$\mathrm{Elovich}\;q_{t}=\frac{1}{\beta }ln\;\left(\alpha \beta \right)+\frac{1}{\beta }\;lnt$$

(6) $$\mathrm{Power}\;\mathrm{function}\;lnq_{t}=lnb+k_{f}\left(lnt\right)$$

(7) $$\mathrm{Intraparticle}\;\mathrm{diffusion}\;q_{t}=c+k_{id}t^{0.5}$$

where *t* represents time, $q_{t}$ is the concentration of DC sorbed at *t* time (mg g^−1^), and $q_{o}$ is the DC sorption at time 0 (mg g^−1^). $k_{1}$ and $k_{2}$ are first- and second-order rate constants and $q_{e}$ represents the amount of DC sorption at equilibrium (mg g^−1^). $k_{2}^{\prime }$ represents the rate constant for pseudo-second-order, *α* represents initial sorption rate (mg g^−1^ min^−1^), *β* represents sorption constant, $k_{f}$ is rate coefficient (mg g^−1^ min^−1^), *b* represents constant, *c* represents diffusion constant, and $k_{id}$ represents apparent diffusion rate constant ((mg g^−1^)^−0.5^).

### 3.8. Isotherm Modeling 

To investigate the sorption capacity of the sorptive materials for DC, nonlinear forms of the Freundlich, Langmuir, Temkin, and Dubinin-Radushkevich isotherm models (Equations (8)–(11), respectively) were applied [74].

(8) $$q_{e}=K_{F}C_{e}^{1/n}$$

(9) $$q_{e}=\frac{Q_{L}C_{e}K_{L}}{1+K_{L}C_{e}}$$

(10) $$q_{e}=\frac{RT}{b}\ln\left(AC_{e}\right)$$

(11) $$q_{e}=q_{D}\exp(-B_{D}{\left[RTln\;\left(1+\frac{1}{C_{e}}\right)\right]}^{2}$$

where, *K_F_* represents the Freundlich sorptive affinity (L g^−^^1^), 1/*n* represents the Freundlich component related to linearity, *Q_L_* represents the Langmuir maximum adsorption capacity (mg g^−^^1^), and *K_L_* represents the Langmuir sorption equilibrium constant (L mg^−^^1^). Binding constant is represented by *A* (L mg^−^^1^), heat of adsorption is denoted by *b*, *q_D_* represents maximum adsorption capacity in g g^−^^1^, while *B_D_* represents the mean free energy, which was further utilized to estimate bonding energy (*E*), as shown in Equation (12):

(12) $$E=\frac{1}{\sqrt{2B_{D}}}$$

### 3.9. Statistical Analyses

All the experiments were conducted in a completely randomized factorial design setting with 3 replications, including blanks. All the data were presented in means of three repeats. Data tabulation, as well calculation of means and standard deviation (SD), were performed using Microsoft© Excel 2016 (Microsoft Inc, Seattle, Washington, USA). The individual data was simulated with kinetics models and nonlinear forms of isotherm models using SigmaPlot 10.0 software (Systat Software Inc., San Jose, California, USA). The nonlinear curve fittings and other graphs were constructed by using SigmaPlot 10.0 software. Pearson’s correlation was calculated using Statistix 8.1 software (tatistix Inc., Florida, USA) to explore the relationship of DC sorption with soil properties. The level of significance for the correlations was set at *p* < 0.05.

The coefficient of determination (*R*^2^) was determined by using Equation (13) to estimate the closeness between the experimental sorption data and the model predicted data.

(13) $$R^{2}=\frac{{\left(q_{em}-\bar{q_{ec}}\right)}^{2}}{\sum {\left(q_{em}-\bar{q_{ec}}\right)}^{2}+{\left(q_{em}-q_{ec}\right)}^{2}}$$

where, *q_ec_* is the calculated amount of DC sorbed (mg g^−1^) at equilibrium, and *q_em_* is the measured amounts of DC sorbed (mg g^−1^) onto soil at equilibrium.

## 4. Conclusions

Sorption and desorption behavior of doxycycline (DC) in three different soils amended with manure and/or mesquite wood-waste-derived biochar (BC) was explored in sorption batch experiments. Soil with pH 7.41, either alone or in combination with manure and/or BC, showed significantly higher sorption of DC as compared to the other two soils with pH of 8.32 and 8.48. DC sorption onto all the tested materials was quick during the initial 200 min, which gradually slowed down and approached equilibrium. Langmuir model predicted maximum sorption capacity was the highest for BMS1 (18.930 mg g^−1^), followed by BMS3 (16.073 mg g^−1^) and BMS2 (15.792 mg g^−1^). Overall, the DC sorption capacity of soil increased by 5.0–6.5-fold with the addition of manure into the soil, whereas it increased up to 10–13-fold when BC was applied in a soil–manure system. Simulation of sorption data with kinetics and isotherm models suggested that electrostatic interactions, H bonding, pore diffusion, and π–π EDA interactions were involved in DC sorption onto the tested materials. Desorption trials revealed that manure application resulted in 67%, 40%, and 41% higher desorption in MS1, MS2, and MS3, as compared respective soils alone, whereas application of BC resulted in 73%, 66%, and 65% lower desorption in BMS1, BMS2, and BMS3, as compared to S1, S2, and S3, respectively. Therefore, application of mesquite wood-waste-derived BC can potentially be used to reduce the mobility of DC in contaminated soil and enhance its productivity through improving physiochemical properties, as well as water and nutrient holding capacity of soil. Further, the findings of this study can be used to formulate efficient and cost-effective strategies to remediate soils contaminated with antibiotic. However, further in-depth studies are needed to investigate the ecotoxic effects and economic value of using mesquite wood waste biochar in real agricultural soils.

## Figures and Tables

**Table 1:** Selected physiochemical properties and proximate attributes of soils (S1, S2, and S3) and manure used in this study.

Property	Unit	S1	S2	S3	Manure
Sand	%	56.40 ± 4.31	73.90 ± 5.15	81.40 ± 5.20	-
Silt	%	17.50 ± 1.45	10.00 ± 0.91	2.50 ± 0.44	-
Clay	%	26.10 ± 3.98	16.10 ± 2.98	16.10 ± 1.12	-
Texture	-	Sandy clay loam	Sandy loam	Sandy loam	-
pH	-	7.41 ± 0.24	8.32 ± 0.21	8.48 ± 0.30	7.72 ± 0.22
EC	dS m^−1^	4.82 ± 0.12	1.42 ± 0.11	2.30 ± 0.02	6.61 ± 0.65
Organic matter	%	0.86 ± 0.09	0.71 ± 0.08	0.67 ± 0.02	10.34 ± 0.84
CEC	cmol kg^−1^	26.39 ± 2.64	18.28 ± 1.98	19.89 ± 0.87	57.29 ± 5.61
CaCO_3_	%	8.55 ± 1.01	9.86 ± 1.80	9.33 ± 0.21	-
Available P	mg kg^−1^	11.89 ± 1.85	8.68 ± 1.35	7.81 ± 0.33	1970.82 ± 18.71
Available K	mg kg^−1^	107 ± 8.15	96 ± 5.41	68 ± 3.64	40.16 ± 5.56
Available Na	mg kg^−1^	34.75 ± 2.87	22.87 ± 1.55	26.43 ± 1.21	46.09 ± 3.67
Doxycycline	μg kg^−1^	N.D.	N.D.	N.D.	N.D.
Moisture	%	1.58 ± 0.01	1.51 ± 0.01	1.43 ± 0.02	13.03 ± 0.28
Volatiles	%	-	-	-	38.72 ± 3.59
Fixed carbon	%	-	-	-	19.29 ± 1.76
Ash	%	-	-	-	28.96 ± 2.87

N.D. = not detected.

**Table 2:** Proximate analyses, surface area, and elemental composition of the mesquite wood waste biomass (BM) and its derived biochar (BC) used in this study.

Property	Unit	BM	BC
Yield	%	–	30.2 ± 4.32
Moisture	%	8.1 ± 0.95	2.10 ± 0.82
Ash	%	1.0 ± 0.16	14.5 ± 1.32
Volatiles	%	72.4 ± 6.71	7.6 ± 0.81
Fixed carbon	%	22.6 ± 1.23	75.8 ± 6.13
Surface area	m^2^ g^−1^	-	196 ± 8.18
Doxycycline	μg kg^−1^	-	N.D.
C	%	-	80.08 ± 6.20
O	%	-	2.49 ± 0.11
H	%	-	1.64 ± 0.05
N	%	-	1.30 ± 0.08
O/C molar ratio	-	-	0.02 ± 0.00
H/C molar ratio	-	-	0.24 ± 0.00
(O + N)/C molar ratio	-	-	0.04 ± 0.00

N.D. = not detected.

**Table 3:** Pearson correlations of various soil properties with doxycycline sorption onto soil alone, manure + soil, and biochar + manure + soil.

		Soil Alone	Manure + Soil	Biochar + Manure + Soil
pH	*r*	−0.999	−0.996	−0.998
	*p*-value	**0.017**	**0.047**	**0.036**
Phosphorus	*r*	0.995	0.997	0.992
	*p*-value	0.060	**0.045**	0.077
CEC	*r*	0.956	0.894	0.963
	*p*-value	0.189	0.295	0.172
CaCO_3_	*r*	−0.866	−0.771	−0.879
	*p*-value	0.332	0.438	0.315
Clay	*r*	0.996	0.967	0.996
	*p*-value	**0.049**	**0.045**	**0.050**
EC	*r*	0.935	0.864	0.944
	*p*-value	0.229	0.335	0.212
Moisture	*r*	0.898	0.959	0.887
	*p*-value	0.288	0.182	0.305
Organic matter	*r*	0.996	0.997	0.997
	*p*-value	**0.050**	**0.047**	**0.045**
Sand	*r*	−0.982	−0.998	−0.977
	*p*-value	0.119	**0.013**	0.136
Silt	*r*	0.915	0.969	0.904
	*p*-value	0.264	0.158	0.280

*r* = Pearson’s correlation coefficients and *p* = probability.

**Table 4:** Parameters obtained from kinetic models for doxycycline sorption onto different soils (pH 7.41 (S1), pH 8.32 (S2), and pH 8.48 (S3)), manure + S1 (MS1), manure + S2 (MS2), manure + S3 (MS3), mesquite wood-waste-derived biochar + MS1 (BMS1), mesquite wood-waste-derived biochar + MS2 (BMS2), and mesquite wood-waste-derived biochar + MS3 (BMS3).

Sorbent	Parameters	S1	S2	S3	MS1	MS2	MS3	BMS1	BMS2	BMS3
First order	*k*_1_	0.001	0.002	0.001	0.001	0.001	0.001	0.001	0.001	0.002
	*R* ^2^	0.308	0.225	0.314	0.665	0.706	0.659	0.520	0.618	0.654
Second order	*k*_2_	−0.008	−0.007	−0.009	0.000	0.000	0.000	0.000	0.000	0.000
	*R* ^2^	0.126	0.154	0.215	0.153	0.181	0.166	0.147	0.172	0.166
Pseudo-first order	$k_{1}^{\prime }$	−0.001	−0.001	−0.001	−0.002	−0.002	−0.002	−0.002	−0.002	−0.002
	*q*_e_	−0.027	−0.414	−0.348	1.447	1.554	1.545	2.067	2.242	2.334
	*R* ^2^	0.245	0.159	0.171	0.684	0.774	0.790	0.746	0.818	0.879
Pseudo-second order	$k_{2}^{\prime }$	7.2 × 10^−4^	3.9 × 10^−2^	1.262	0.983	1.319	2.474	1.237	0.756	0.880
	*q*_e_	1.870	0.022	7.0 × 10^−4^	9.2 × 10^−4^	6.7 × 10^−4^	3.5 × 10^−4^	7.1 × 10^−4^	1.1 × 10^−3^	1.0 × 10^−3^
	*h*	2.5 × 10^−3^	1.9 × 10^−5^	6.2 × 10^−7^	8.1 × 10^−7^	5.9 × 10^−7^	3.1 × 10^−7^	6.3 × 10^−7^	1.0 × 10^−7^	8.9 × 10^−7^
	*R* ^2^	0.397	0.103	0.105	0.104	0.104	0.104	0.104	0.104	0.104
Elovich	*a*	0.173	0.150	0.142	0.910	0.886	0.875	1.897	1.793	1.763
	*β*	0.282	0.243	0.251	0.880	1.164	1.208	1.914	2.207	2.611
	*R* ^2^	0.977	0.992	0.981	0.997	0.993	0.997	0.965	0.997	0.992
Intraparticle diffusion	*k_id_*	0.034	0.029	0.028	0.163	0.167	0.165	0.330	0.331	0.339
	*c*	−0.039	−0.017	−0.054	0.683	0.222	0.152	1.485	0.698	0.023
	*R* ^2^	0.984	0.930	0.958	0.942	0.971	0.966	0.871	0.956	0.967
Power function	*k_f_*	0.142	0.185	0.123	0.280	0.293	0.302	0.383	0.378	0.396
	*b*	−1.507	−2.087	−1.715	−0.255	−0.501	−0.610	−0.155	−0.263	−0.516
	*R* ^2^	0.830	0.751	0.925	0.962	0.958	0.926	0.888	0.947	0.926

Note: $k_{1}$ and $k_{2}$ are first- and second-order rate constant, $q_{e}$ represents the amount of DC sorption at equilibrium (mg g^−1^), $k_{1}^{\prime }$ and $k_{2}^{\prime }$ represent the rate constant for pseudo-first and pseudo-second-order, *α* represents initial sorption rate (mg g^−1^ min^−1^), *β* represents sorption constant, $k_{f}$ is rate coefficient (mg g^−1^ min^−1^), *b* represents constant, *c* represents diffusion constant, and $k_{id}$ represents apparent diffusion rate constant ((mg g^−1^)^−0.5^).

**Table 5:** Parameters obtained from nonlinear isotherm models for doxycycline sorption onto different soils (pH 7.41 (S1), pH 8.32 (S2), and pH 8.48 (S3)), manure + S1 (MS1), manure + S2 (MS2), manure + S3 (MS3), mesquite wood-waste-derived biochar + MS1 (BMS1), mesquite wood-waste-derived biochar + MS2 (BMS2), and mesquite wood-waste-derived biochar + MS3 (BMS3).

Isotherms	Parameters	S1	S2	S3	MS1	MS2	MS3	BMS1	BMS2	BMS3
Langmuir	*Q_L_* (mg g^−1^)	1.210	1.059	1.083	6.497	6.708	6.604	18.930	15.792	16.073
	*K_L_* (L g^−1^)	0.548	0.159	0.114	1.006	0.388	0.357	2.049	2.050	1.818
	*R* ^2^	0.988	0.995	0.994	0.984	0.992	0.992	0.995	0.999	0.997
Freundlich	*K_F_* (L g^−1^)	0.494	0.232	0.194	2.582	1.919	1.803	13.263	9.518	9.266
	*1/n*	0.253	0.382	0.417	0.291	0.366	0.377	0.618	0.540	0.539
	*R* ^2^	0.949	0.961	0.973	0.916	0.928	0.937	0.996	0.992	0.993
Temkin	*b* (J mol^−1^)	1.23 × 10^4^	1.14 × 10^4^	1.20 × 10^4^	2.71 × 10^3^	2.93 × 10^3^	3.03 × 10^3^	9.35 × 10^2^	1.04 × 10^3^	1.53 × 10^3^
	*A* (L g^−1^)	15.078	2.430	2.213	48.203	27.544	26.895	84.938	76.535	228.050
	*R* ^2^	0.968	0.980	0.952	0.904	0.787	0.777	0.907	0.906	0.725
Dubinin–Radushkevich	*q_D_* (mg g^−1^)	1.044	0.856	0.825	5.707	5.487	5.313	13.833	12.765	12.777
	*E* (kJ g^−1^)	8.0 × 10^−4^	6.6 × 10^−3^	9.1 × 10^−3^	3.0 × 10^−4^	9.0 × 10^−4^	1.0 × 10^−3^	1.0 × 10^−3^	1.0 × 10^−4^	1.0 × 10^−3^
	*R* ^2^	0.863	0.881	0.873	0.951	0.962	0.960	0.974	0.984	0.984

Note: *Q_L_* represents the Langmuir maximum adsorption capacity, *K_L_* represents the Langmuir sorption equilibrium constant, *K_F_* represents the Freundlich sorptive affinity, 1/n represents the Freundlich component related to linearity, *A* is the binding constant, b is the heat of adsorption, *q_D_* represents maximum adsorption capacity, *B_D_* represents the mean free energy, and *E* the bonding energy.

## Data Availability

Not applicable.
